# The Power of Databases in Unraveling the Nutrition–Health Connection

**DOI:** 10.3390/nu17101725

**Published:** 2025-05-20

**Authors:** Andreu Farran-Codina, Mireia Urpí-Sardà

**Affiliations:** 1 Departament de Nutrició, Ciències de l’Alimentació i Gastronomia, Facultat de Farmàcia i Ciències de l’Alimentació, Campus de l’Alimentació de Torribera, Universitat de Barcelona (UB), 08921 Santa Coloma de Gramenet, Spain; murpi@ub.edu; 2 Institut de Recerca en Nutrició i Seguretat Alimentària (INSA-UB), Campus de l’Alimentació de Torribera, Universitat de Barcelona (UB), 08921 Santa Coloma de Gramenet, Spain; 3 Centro de Investigación Biomédica en Red de Fragilidad y Envejecimiento Saludable (CIBERFES), Instituto de Salud Carlos III, 28029 Madrid, Spain

Human activities across different sectors produce large volumes of relevant nutritional information. These data, which are derived from various sources (food analysis, consumption surveys, health monitoring, or environmental assessments) are systematically gathered and organized in databases. These databases are a crucial tool for contemporary nutrition research. By structuring and integrating these varied datasets, researchers can identify patterns, associations, and trends that enhance our understanding of the intricate link between nutrition and health [1]. The increasing volume and complexity of nutrition data necessitate advanced analytical approaches, and in this context, artificial intelligence (AI) is emerging as a transformative force. Techniques like text mining, a branch of AI focused on extracting insights from textual data, are proving invaluable for leveraging the wealth of information contained within nutritional databases and related sources.

Consequently, databases are vital for organizing knowledge, enabling the creation of new data that enhances and expands scientific understanding. They have a role in multiple areas, such as analyzing nutrient consumption and food contaminants, or assessing how dietary patterns affect health outcomes. The elaboration and validation of dietary guidelines and the formulation of evidence-based policies require the use of extensive and complex nutritional and health databases because of the growing complexity of dietary patterns, food supply chains, and the relationship between eating habits and metabolic diseases [2]. To further enhance the power of these databases, machine learning (ML), another key area within AI, is playing an increasingly significant role [3]. ML algorithms can analyze vast datasets within nutritional databases to uncover intricate relationships and build predictive models that go beyond traditional statistical methods.

One of the most exciting and promising uses of nutritional databases is their combination with metabolomics data, allowing scientists to study how our bodies biochemically react to various dietary components. Metabolomics is key to discovering objective food intake biomarkers, which improve upon self-reported dietary data [4]. While metabolomic profiling is advancing biomarker identification and applications in nutrition research are growing, the field faces challenges. These include the crucial need for validated biomarkers and specialized databases of food-derived metabolites [4]. In addition, the application of ontologies like the Food-Biomarker Ontology (FOBI) facilitates the connection between food intake data and metabolomic profiles, leading to more precise dietary assessments [5]. Metabolomics is especially important in precision nutrition, where understanding individual metabolic responses is crucial for providing personalized dietary advice. By merging metabolomics databases with traditional food composition databases, researchers can more accurately predict the impact of diet on health and disease prevention [6]. Recent advancements in AI and ML are further enhancing this field, enabling the creation of predictive models that customize dietary recommendations to meet individual needs [7].

Databases are also driving innovation in food analysis and description. Standardizing food composition datasets across different regions and dietary traditions is critical for improving AI-driven applications in food recognition and dietary assessment. A great example is the FAO/INFOODS initiative, which has worked to harmonize food composition tables, ensuring data consistency on a global scale [8]. In Europe, the international association EuroFIR provides standardized and validated information on the composition of foods marketed in different European countries [9]. These efforts not only facilitate research but also boost AI-based tools designed for personalized nutrition and health monitoring. Recent studies have shown how AI and ML are transforming clinical nutrition, improving decision-making, predicting nutritional deficiencies, and optimizing dietary interventions in critical care settings [10].

This Special Issue dedicated to “Databases, Nutrition and Health” gathers diverse studies united by their reliance on databases to address critical questions across the spectrum of nutrition and health. As the reader will find within these pages, the effective use and development of databases are fundamental for progress—from shaping public health policies and interventions using large-scale surveillance insights to enhancing clinical decision-making and even ensuring food safety. The contributions herein exemplify these crucial and diverse roles, tackling specific challenges and showcasing innovations across various domains. For instance, the importance of robust databases for public health policy and surveillance is clearly highlighted by Al Jawaldeh et al., who reviews national nutrition surveillance systems in the Eastern Mediterranean Region, identifying strengths, weaknesses, and the critical need for data integration and standardization, especially in fragile settings. Bridging the gap between different food cultures and technologies, Bianco et al. address the intricate challenges of adapting and harmonizing food composition databases (FCDBs)—in their case, Italian data for the US Nutrition5k dataset—essential for training reliable AI-driven food image recognition tools. The power of large-scale epidemiological databases is demonstrated by Li et al., using NHANES data to reveal how adequate dietary fiber intake might mitigate the detrimental impact of environmental contaminants like blood lead on dyslipidemia risk among US adults. In the clinical sphere, Garcia-Arenas et al. underscore the necessity for specialized, up-to-date databases by creating and applying a database of special low-protein foods (SLPFs) to optimize dietary management for patients with inborn errors of metabolism, showing how SLPF intake impacts dietary patterns and biochemical profiles. Expanding into gene–diet interactions, Lee et al. leverage longitudinal cohort data (KoGES) to show how food group intake (like fruits, meat, or beverages) can significantly modify the risk of insomnia associated with specific *CLOCK* gene polymorphisms, highlighting sex-specific differences. Finally, offering a critical perspective on the evidence base itself, Kamioka et al. assess the methodological quality of systematic reviews supporting ‘Foods with Function Claims’ (FFC) in Japan, finding significant deficiencies in aspects like protocol registration, search strategies, and risk of bias assessment, thus emphasizing the importance of rigorous methodology when using databases for regulatory claims.

Looking ahead, the evolution of nutritional databases is anticipated to feature more sophisticated algorithms for data analysis, better procedures to ensure data quality, improved accessibility for researchers and policymakers, and the better integration of real-time health monitoring tools. As datasets grow and become more interconnected, the collaboration between nutrition science, technology, and public health will strengthen, leading to more accurate dietary recommendations and effective health interventions. The contributions to this Special Issue highlight the essential role of databases in advancing nutrition research, underscoring the need for continuous refinement, harmonization, and innovation in the field.
